# Radiographic Evolution of Adult Native Hip Septic Arthritis: A Case Report

**DOI:** 10.5704/MOJ.2507.018

**Published:** 2025-07

**Authors:** JY Low, YP Khor

**Affiliations:** Department of Orthopaedic Surgery, Ng Teng Fong General Hospital, Singapore

**Keywords:** hip, septic arthritis, radiograph

## Abstract

A 31-year-old man presented with abscesses of the left iliacus muscle, adductor muscles and left hip septic arthritis. Following surgical debridement and antibiotics, he remained infection free at 3 years. We present the radiographic evolution of the changes in his left hip . Despite destruction of the hip during initial follow-up, there was remoulding of the proximal femur with changes of avascular necrosis over a 3-year period with good hip function. Hip septic arthritis may result alarming radiographic changes during initial follow-up. Some patients may continue to improve clinically and radiographically in the short term.

## Introduction

Adult native hip joint septic arthritis is rare. The hip joint is the second most common large joint, after the knee affected in adult native joint septic arthritis^1^.

Most of the available information on native hip joint septic arthritis management discusses the efficacy of various treatment modalities. These include medical therapy with antibiotics or in combination with surgical therapy. Surgical options include joint lavage and, one or two stage joint replacement. In cases where the joint cartilage is found to be destroyed, surgeons may advise joint resection and arthroplasty. There is little available in literature describing the radiographic sequelae of patients with hip septic arthritis.

We found only one case report describing serial radiographs in a patient with septic hip arthritis^2^. We present an interesting series of radiographs of left hip septic arthritis in a man with preservation of native joint and good functional outcome at three years. The patient has been informed that the data concerning this case will be submitted for publication and he has provided consent.

## Case Report

A 31-year-old man with a history of hyperthyroidism presented with a 2-week history of left groin pain. He had a fever of 39.5^o^C and tachycardia. He had left iliac fossa pain and inability to rotate the left hip.

**Fig. 1:**
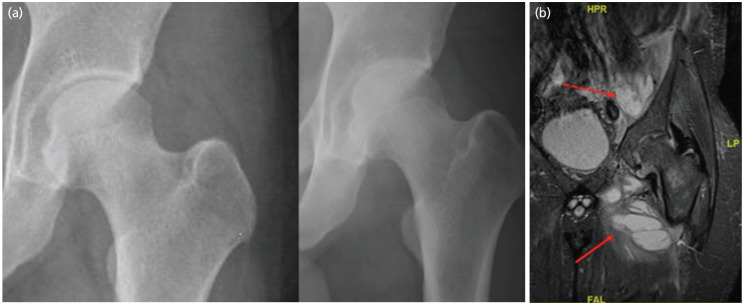
(a) Pelvis AP Radiographs two weeks apart showing acute loss of left hip joint space. (b) MRI (coronal section) showing iliacus abscess (dotted arrow) and adductor abscess (solid arrow). Evidence of destruction of femoral head cartilage and marrow enhancement.

Blood investigations showed raised white blood cell count (WBC) of 16.84 x 109/L, erythrocyte sedimentation rate (ESR) of >80mm/hour and C-reactive protein (CRP) of 334.7mg/L. His radiographs showed acute reduction in the left hip joint space compared to radiographs taken at the onset of symptoms (Fig. 1a).

Computer Tomography (CT) scan of the pelvis showed a 4 x 2.5 x 4cm abscess of the left iliacus muscle. Magnetic Resonance Imaging (MRI) showed left hip joint effusion with marrow enhancement of the left femoral head. There was a 10.0 x 7.6 x 4.0cm intramuscular abscess of the left adductor muscles (Fig. 1b).

He underwent surgery to drain the adductor abscess and washout the left hip joint. The iliacus muscle abscess was not drained surgically. The left hip joint arthrotomy was performed through the Smith-Peterson approach. Purulent fluid was encountered, and the femoral head cartilage appeared normal. The joint was washed and debrided of its synovium. A medial thigh incision was made to drain the abscess of the adductor magnus.

Blood and tissue cultures grew Methicillin-Sensitive Staphylococcus aureus. The patient was managed with intravenous cefazolin for six weeks followed by oral cefalexin for 12 weeks.

His workup for infection risk factors were negative for diabetes mellitus, Hepatitis B and C, retroviral disease and valvular vegetations. A repeat CT scan at two weeks showed resolution of the iliacus muscle abscess.

He was placed on continuous passive motion of the hip immediately after surgery. Weight bearing was allowed as tolerated with crutches and he was discharged with outpatient physiotherapy and intravenous antibiotics.

**Fig. 2:**
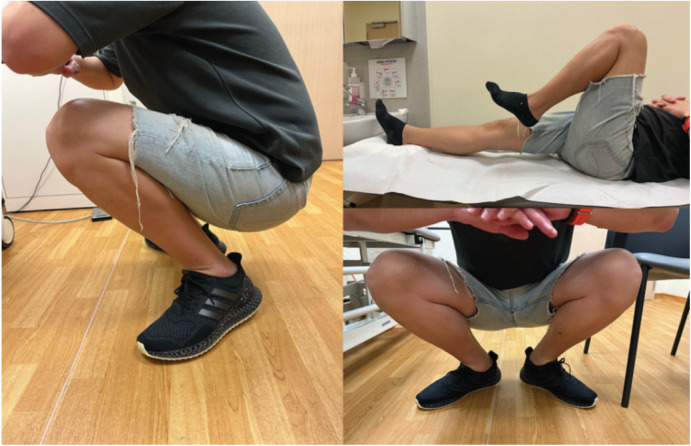
Serial radiographs at (a) nine weeks and (b) four months showing destruction of left hip joint with flattening of the femoral head. (c) One-year radiographs showing early avascular necrosis of femoral head and cystic changes at the acetabulum. Femoral head had remodelled against the acetabular socket. (d) Three-year follow-up radiographs without further collapse of femoral head.

At the nine-week review, radiograph revealed destruction of the femoral head (Fig. 2a). He was warned of the potential need for arthroplasty. At four months, he was walking with a stick and pain improved. Blood results showed normal WBC and CRP. However, radiographs show destruction of the left hip joint (Fig. 2b). He was cleared to drive a private hire vehicle at six months and was able to work.

At one-year post-surgery, there was further clinical improvement with his hip range of motion. Radiographs showed early signs of avascular necrosis (Fig. 2c). Interestingly, the femoral head had appeared to have remodelled against the shape of the acetabulum.

**Fig. 3:**
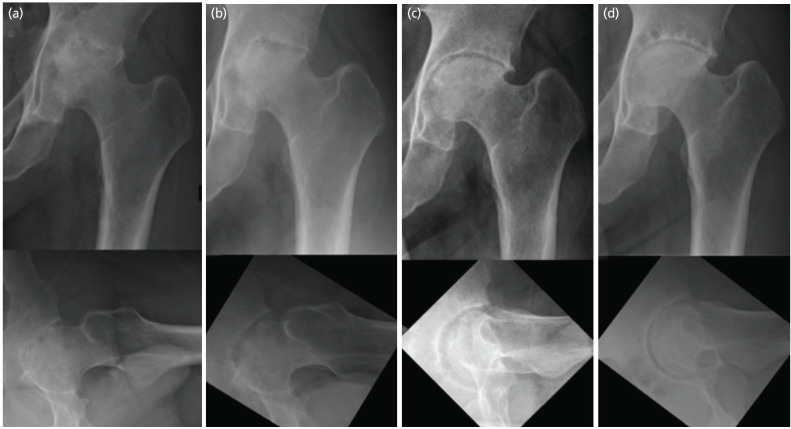
Clinical images showing patient’s function at three years follow-up.

At three years post-op, despite a 1cm leg length discrepancy, he did not require walking aids, or a shoe raise and described no difficulties with activities of daily living. He could perform a full squat without pain (Fig. 3) and cycles 10km a day.

Nonetheless, radiographs showed progression of secondary osteoarthritis of the left hip without collapse of the femoral head (Fig. 2d).

## Discussion

Adult native hip joint septic arthritis is rare with worldwide yearly incidences of approximately 4 to 10 per 100000 patients^3^. The aetiologies include haematogenous spread and direct spread from trauma. The most common pathogen isolated from hip septic arthritis is Staphylococcus aureus^3^ with risk factors including rheumatoid arthritis, diabetes mellitus, recent instrumentation and intravenous drug users.

The difficulty in prescribing an algorithm for management of adult native hip septic arthritis stems from the rarity of the disease and variability in their clinical presentation. The hip joint being a deeply located joint is not readily accessible for assessment of effusion and signs of infection may be subtle.

Patient can present acutely with marked evidence of systemic sepsis or a more indolent course leading to late diagnosis. Nonetheless, the principles and goals of management for septic arthritis are the same. These include, prompt diagnosis, early identification of a pathogen for delivery of targeted antibiotics, eradication of infection, preservation of joint and function of the patient.

Most of the body of literature discussing hip joint septic arthritis focus on eradication of infection by means of antibiotics, serial aspiration, arthroscopic or open washout, resection arthroplasty and joint replacement. With presence of joint destruction, some patients are offered joint replacement after resection arthroplasty, which has close to 93% of success rate^4^. Despite successful eradication of infection following surgery and antibiotic therapy, approximately 9.8% of patients have recurrence of infection within two years after surgery^5^.

We present an interesting radiographic progression of the disease in our patient who maintained a spherical femoral head at three years with good function despite initial destruction seen at four months. We came across one other case report showing a serial change in radiographs^2^ over a six-week period. The destruction seen on radiographs was deemed unsalvageable and their patient went on to have a two-stage joint replacement. Our report presents a longer duration of radiographic follow-up after the destructive changes seen at six weeks and three months. As our patient was free from infection recurrence and had no pain, we were able to perform serial monitoring rather than a hip replacement. We do not know if the patient from the other case report required surgery due to symptoms as this was not described in their report.

Despite overt joint destruction on initial radiographs, there may be a subset of patients who continue to improve functionally over time. Surgeons need not rush into performing arthroplasty based on severe radiographic changes if infection appears to have been eradicated with good clinical function.
